# Losartan-Induced Sprue-Like Enteropathy Presenting With Isolated Ileal Villous Atrophy: A Report of a Rare and Atypical Case

**DOI:** 10.7759/cureus.108981

**Published:** 2026-05-16

**Authors:** Carlos Berrocal, Juanita Vasquez Villada, Pablo Galindo Orrego, Isabella Libreros Mejia, Diana Marcela Delgadillo Arco, Alejandro Mitsuaki Suguimoto Erasso, Mariana Caballero Lagares, Samuel Enrique Solano Álvarez, Víctor Alonso Carrascal López

**Affiliations:** 1 Internal Medicine, Universidad del Valle, Cali, COL; 2 Internal Medicine, Division of Gastroenterology, Universidad del Valle, Cali, COL; 3 Medicine, Universidad Libre, Cali, COL; 4 Medicine, Universidad del Valle, Cali, COL; 5 Pathology, Clínica Imbanaco Grupo Quirónsalud, Cali, COL; 6 Medicine, Hospital Universitario San Vicente de Paúl, Medellín, COL; 7 Medicine, Clínica Fundación Amigos de la Salud, Montería, COL

**Keywords:** angiotensin receptor antagonists, chronic watery diarrhea, intestinal villous atrophy, losartan, malabsorption syndrome

## Abstract

Sprue-like enteropathy associated with angiotensin II receptor blockers (ARBs) is a rare cause of chronic diarrhea and malabsorption, typically characterized by villous atrophy and intraepithelial lymphocytosis mimicking celiac disease. Although olmesartan is the most frequently implicated agent, cases associated with other ARBs, including losartan, have increasingly been recognized.

A 61-year-old woman with hypertension treated with losartan for three years presented with a three-month history of abdominal pain and chronic diarrhea. Upper endoscopy revealed a macroscopically and histologically normal duodenum. Colonoscopy demonstrated macroscopically normal ileal mucosa; however, ileal biopsies showed villous atrophy and marked intraepithelial lymphocytosis. Celiac serology was negative, infectious causes were excluded, and vitamin B12 deficiency was documented. In the setting of chronic exposure to losartan and after exclusion of alternative etiologies, ARB-associated sprue-like enteropathy was suspected. Losartan was discontinued and replaced with amlodipine, resulting in complete clinical resolution.

This case highlights an atypical presentation of losartan-induced sprue-like enteropathy with isolated ileal involvement and a histologically normal duodenum. Clinicians should consider this entity in patients presenting with chronic diarrhea and seronegative villous atrophy, even in the absence of duodenal involvement.

## Introduction

Enteropathy associated with angiotensin II receptor blockers (ARBs) is an uncommon cause of chronic diarrhea and malabsorption that has been increasingly recognized over the past decade. This entity is characterized by severe gastrointestinal symptoms, including persistent diarrhea, weight loss, and histological alterations of the small intestine, mainly villous atrophy and intraepithelial lymphocytosis, findings that may mimic celiac disease [1,2].

Olmesartan is the drug most frequently implicated; however, isolated cases associated with other ARBs, including losartan, have also been reported. Due to its low frequency and its clinical and histological resemblance to other enteropathies, particularly celiac disease, this condition often poses a diagnostic challenge, especially in patients with seronegative villous atrophy or persistent symptoms despite a gluten-free diet. Recognition of this entity is clinically important because delayed diagnosis may lead to prolonged symptoms, unnecessary diagnostic testing, and avoidable dietary restrictions.

We present the case of a patient with losartan-induced sprue-like enteropathy characterized by isolated ileal villous atrophy and a histologically normal duodenum, an uncommon presentation within the spectrum of this condition.

## Case presentation

A 61-year-old woman with a history of hypertension treated with losartan 50 mg orally once daily for the past three years, and type 2 diabetes mellitus managed with dapagliflozin 10 mg orally once daily, presented with a three-month history of intermittent abdominal pain localized to the flanks and hypogastrium. Her symptoms were associated with changes in bowel habits, characterized by loose to watery stools occurring up to three times per day. Notably, the patient had previously been treated with metformin; however, this medication had been discontinued one month before presentation without any improvement in her symptoms.

On physical examination, the patient was alert and hemodynamically stable. Abdominal examination revealed a soft, non-tender abdomen on deep palpation with no palpable masses, organomegaly, or signs of peritoneal irritation.

**Figure 1:**
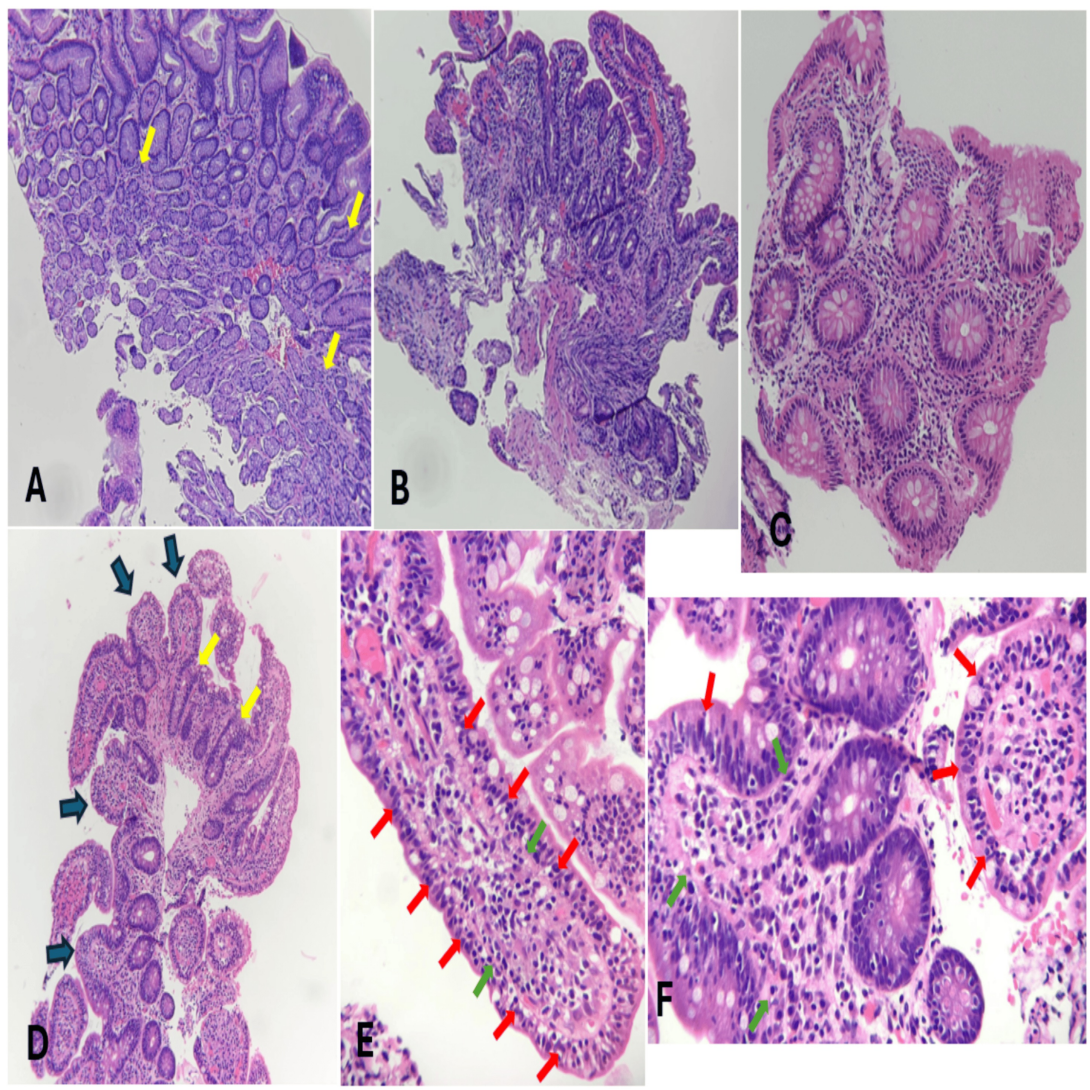
Histopathological findings on hematoxylin and eosin (H&E) staining (A) (10×) Antral gastric mucosa with preserved glandular architecture and mild superficial mononuclear inflammatory infiltrate within the lamina propria (yellow arrows), without evidence of acute inflammatory activity. (B) (10×) Duodenal mucosa without significant histopathological abnormalities. No erosions, ulceration, granulomas, or infectious microorganisms are identified. (C) (10×) Colonic mucosa with preserved crypt architecture and no significant inflammatory or structural alterations. (D) (4×) Ileal mucosa showing marked villous atrophy with villous blunting (blue arrows) and crypt hyperplasia (yellow arrows). (E) (40×) Ileal mucosa demonstrating shortened villi with architectural distortion and relative preservation of crypt structures, with intraepithelial lymphocytic infiltration (red arrows) and inflammatory infiltrate in the lamina propria (green arrows). (F) (40×) Ileal mucosa revealing epithelial regenerative changes in the crypts and increased intraepithelial lymphocytes (red arrows), along with a mild lymphoplasmacytic infiltrate in the lamina propria (green arrows).

**Figure 2:**
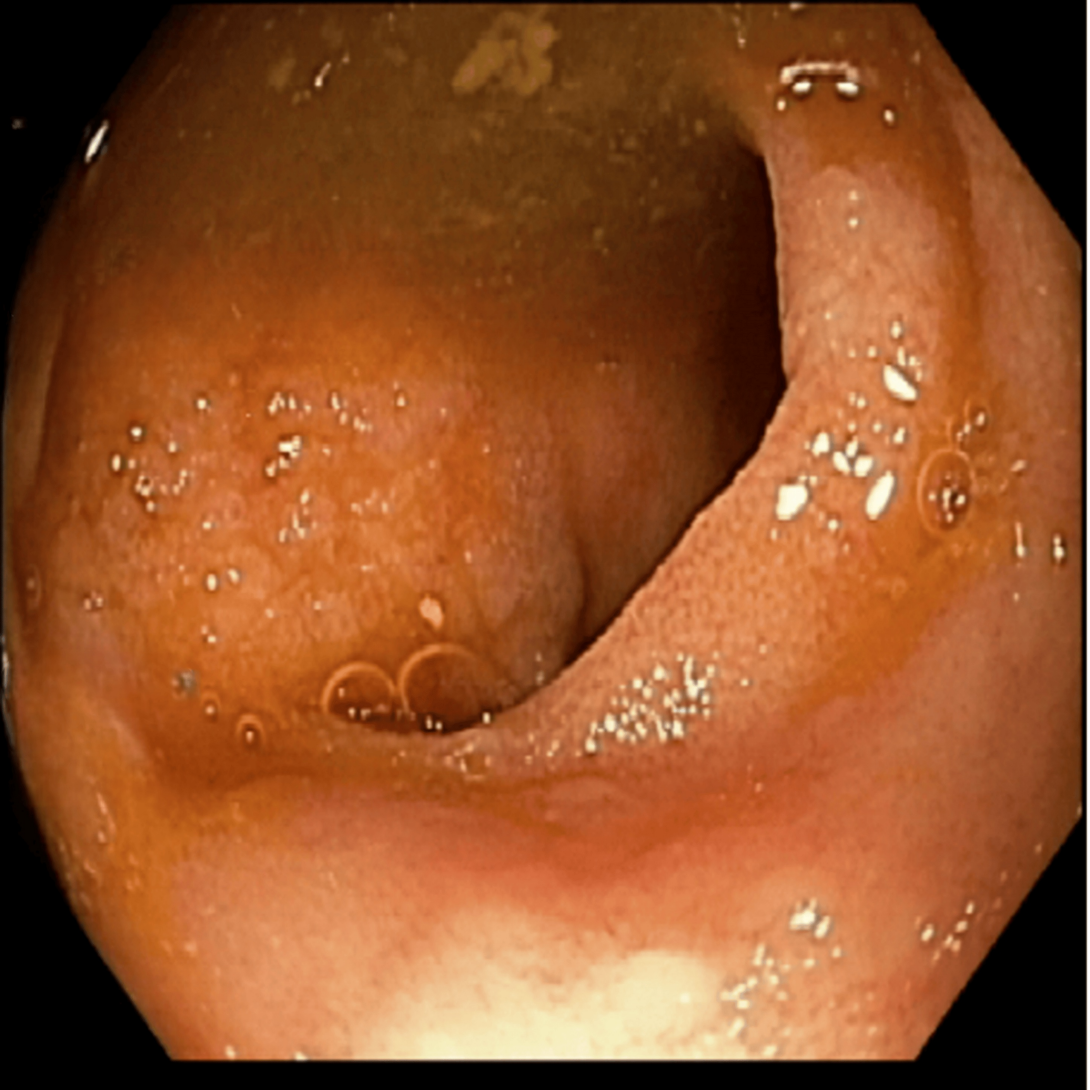
Colonoscopic appearance of the terminal ileum Representative endoscopic views of the terminal ileum demonstrate preserved mucosal architecture with normal villous pattern, intact vascular markings, and no evidence of erosions, ulceration, or inflammatory changes, consistent with a macroscopically normal ileal mucosa.

An esophagogastroduodenoscopy was subsequently performed, revealing macroscopically and histologically normal duodenal mucosa (Figure 1B). Histopathological examination of gastric biopsies demonstrated chronic non-atrophic gastritis without evidence of *Helicobacter pylori* infection (Figure 1A). Total colonoscopy with adequate bowel preparation revealed colonic diverticulosis without endoscopic evidence of inflammation. The terminal ileum appeared macroscopically normal on ileoscopy (Figure 2). However, histopathological evaluation of ileal biopsies demonstrated villous shortening with a reduced villous-to-crypt ratio. In addition, marked intraepithelial lymphocytosis was identified within the surface epithelium, with approximately 55 intraepithelial lymphocytes per 100 enterocytes (Figures 1D-1F). The lamina propria showed increased lymphoplasmacytic infiltration along with up to 5 eosinophils/HPF. Colonic biopsies demonstrated preserved mucosal architecture with up to 5 eosinophils/HPF and no other significant histopathological abnormalities (Figure 1C).

In light of these findings, serological testing for celiac disease, including anti-tissue transglutaminase IgA antibodies, was performed and returned negative, with normal total IgA levels (Table 1). The discordance between the presence of ileal villous atrophy and negative serological results, particularly in the context of a histologically normal duodenum, prompted further evaluation to exclude alternative etiologies.

**Table 1:** Laboratory findings tTG: tissue transglutaminase; IgA: immunoglobulin A

Test	Result	Reference values
tTG (U/mL)	<2.00	Negative <20, Positive ≥20
Fecal calprotectin (µg/g)	71.80	Normal <50, Borderline 50-120, Elevated >120
Serum IgA (mg/dL)	288	69-517
Vitamin B12 (pg/mL)	169.87	187-883
Sudan III (stool)	Negative	Negative
Stool ova and parasite examination	Negative for protozoa, helminths, and cysts	Negative
Stool leukocytes (Wright stain)	Negative	Negative
Fecal occult blood test	Positive	Negative
Stool pH	7.0	6.5-7.5

Subsequent stool studies were negative for parasitic infection. Stool pH was within the normal range, suggesting the absence of carbohydrate malabsorption, while Sudan III staining was negative, indicating no evidence of significant fat malabsorption. Fecal calprotectin levels were borderline, fecal occult blood testing was positive, and vitamin B12 deficiency was documented (Table 1). A complete blood count revealed no evidence of leukocytosis or left shift.

Given the patient’s chronic exposure to an ARB, specifically losartan, ARB-associated enteropathy was considered. Consequently, losartan was discontinued and replaced with amlodipine 5 mg orally once daily as an alternative antihypertensive agent.

Following withdrawal of losartan, the patient exhibited progressive clinical improvement, with complete resolution of diarrhea within four weeks. Based on the favorable clinical response after discontinuation of the suspected drug and the exclusion of other potential etiologies, a diagnosis of losartan-induced sprue-like enteropathy was established.

## Discussion

Enteropathy associated with ARBs, also referred to as ARB-associated sprue-like enteropathy, is a clinical entity characterized by severe chronic diarrhea, significant weight loss, and malabsorption, which may occasionally require hospitalization [1,2].

ARBs are a widely prescribed class of antihypertensive agents that act by blocking angiotensin II binding to AT1 receptors, thereby inducing vasodilation and lowering blood pressure [3]. In recent years, their use has been increasingly associated with enteropathy, defined as inflammatory and/or atrophic injury of the small intestine secondary to chronic exposure to these agents, which may mimic celiac disease or other malabsorptive disorders [4,5].

Olmesartan is the most frequently implicated drug; however, sporadic cases associated with other ARBs have been reported. In a systematic review by Kamal et al. (2019), which included 248 cases of ARB-associated sprue-like enteropathy, 94% were attributed to olmesartan, whereas only 0.8% were associated with losartan [6]. Similarly, Schiepatti et al. (2024) identified 183 cases across 94 studies, with olmesartan accounting for approximately 90% of cases. Other ARBs were less commonly involved, including valsartan (3.6%), telmisartan (2.2%), irbesartan (1.6%), and losartan (three cases, 1.6%). The median duration of exposure before symptom onset was 36 months [7].

The pathophysiology appears to be mediated by an idiosyncratic, immune-related mechanism, although it remains incompletely understood. A leading hypothesis involves two angiotensin receptor subtypes - AT1 and AT2 - expressed throughout the gastrointestinal tract. AT1 receptors contribute to intestinal homeostasis, whereas AT2 receptors are associated with epithelial apoptosis. As losartan acts as a selective competitive antagonist of AT1 receptors, its blockade may favor angiotensin II binding to AT2 receptors, thereby enhancing proapoptotic signaling and contributing to villous atrophy and intraepithelial lymphocytosis [8].

In addition, ARBs may mimic the innate effects of gluten observed in celiac disease by inducing overexpression of interleukin-15 (IL-15) and its receptor (IL-15R) in the intestinal epithelium. This promotes recruitment and activation of cytotoxic CD8+ T lymphocytes responsible for mucosal injury. This process may be further exacerbated by disruption of the tight junction protein ZO-1, leading to impaired intestinal barrier integrity, as well as by dysregulated immune responses in which increased FoxP3+ regulatory cells fail to suppress inflammation, possibly due to interference from IL-15 signaling [9]. However, these findings have not been consistently reproduced across all ARBs, suggesting that susceptibility may depend on drug-specific mechanisms and individual host factors [8].

Another proposed mechanism involves T cell-mediated epithelial injury secondary to altered mucosal immune homeostasis in predisposed individuals. In a systematic review of ARB-associated sprue-like enteropathy, 71.4% of patients carried HLA-DQ2/DQ8 haplotypes, supporting a celiac-like susceptibility despite negative serology in 98.8% of cases [6].

Clinically, this condition typically affects older adults, with a mean age of approximately 70 years, and shows a female predominance. The most common manifestation is persistent watery diarrhea, reported in up to 97% of cases, followed by significant weight loss (84%, median approximately 13 kg), vomiting, and, in some cases, electrolyte disturbances and renal dysfunction secondary to dehydration [7].

Diagnosis is primarily one of exclusion and requires a high index of suspicion, particularly in patients receiving olmesartan or other ARBs who present with chronic diarrhea and villous atrophy without response to a gluten-free diet [10]. It is essential to exclude other causes of seronegative villous atrophy, including seronegative celiac disease (HLA-DQ2/DQ8 positivity and response to a gluten-free diet), autoimmune enteropathy, common variable immunodeficiency (CVID), infections (e.g., Giardia, Whipple disease), intestinal lymphoma, Crohn’s disease, and tropical sprue [11].

Endoscopic evaluation with duodenal biopsies typically demonstrates villous atrophy with intraepithelial lymphocytosis, often accompanied by eosinophilic infiltration, while preserving neuroendocrine, Paneth, and goblet cells. Although these findings are most commonly described in the duodenum, ARB-associated enteropathy may involve other segments of the gastrointestinal tract. Schiepatti et al. (2024) [7] reported villous atrophy in 89% of patients with additional involvement of the gastric and colonic mucosa. Gastric manifestations may include lymphocytic or eosinophilic gastritis, whereas colonic findings can resemble microscopic colitis, suggesting a broader spectrum of injury beyond that observed in classical celiac disease.

Diagnostic confirmation relies on clinical improvement following discontinuation of the ARB, with progressive resolution of diarrhea and weight recovery [11]. In patients undergoing follow-up endoscopy, histological normalization of the intestinal mucosa after drug withdrawal has also been documented [6,12]. Re-exposure to the offending agent - although not recommended for ethical reasons - typically leads to symptom relapse, underscoring the importance of avoiding future use [6,13]. Therefore, early recognition and permanent discontinuation of the implicated ARB remain the cornerstone of management and are generally associated with an excellent prognosis.

There is no evidence supporting the routine use of immunosuppressive therapy, systemic corticosteroids, or specific diets such as a gluten-free diet, as clinical and histological recovery are usually complete after drug withdrawal [14]. In rare cases of severe disease or persistent symptoms despite discontinuation, adjunctive therapy with topical corticosteroids such as budesonide has been reported; however, this approach is based on limited evidence and is not considered standard treatment [11].

In our patient, who had been receiving losartan, the clinical presentation was consistent with ARB-associated sprue-like enteropathy. A comprehensive evaluation excluded infectious and other causes of malabsorption; stool examination showed normal pH, and celiac serology (anti-tissue transglutaminase antibodies) was negative. Following discontinuation of the drug, a marked improvement in gastrointestinal symptoms was observed.

It is noteworthy that the patient had previously been treated with metformin, a drug known to cause gastrointestinal symptoms; however, its prior discontinuation did not result in clinical improvement, further supporting the role of losartan in the development of this condition.

A review of the literature identified three reported cases of losartan-associated sprue-like enteropathy (Table 2), with patient ages ranging from 59 to 73 years. In the case described by Negro et al. (Italy, 2015), the patient presented with severe diarrhea and significant weight loss [15]. In the report by van Gils et al. (Netherlands, 2017), celiac disease was initially suspected due to persistent symptoms despite a gluten-free diet [16]. Essien et al. (United States, 2021) described a case associated with cutaneous ulcerative manifestations [17].

**Table 2:** Reported cases of losartan-associated sprue-like enteropathy: clinical, histological, and therapeutic characteristics Note: *Intraepithelial lymphocytosis is expressed as the number of lymphocytes per 100 enterocytes.

Author (year, country)	Sex/age (years)	Main symptoms	Intestinal location	Histological findings	Nutritional findings	Celiac serology	Treatment	Outcome
Negro et al. (2015, Italy) [15]	Male/67	Severe chronic diarrhea	Duodenum	Sprue-like villous atrophy	Malabsorption (significant weight loss, hypoalbuminemia, anemia, multiple micronutrient deficiencies (including vitamins A, D, and B6)	Negative	Discontinuation of losartan	Clinical resolution
van Gils et al. (2017, Netherlands) [16]	Male/73	Persistent diarrhea	Duodenum	Villous atrophy with celiac-like changes	Malabsorption (steatorrhea and significant weight loss)	Negative	Discontinuation of losartan	Clinical resolution
Essien et al. (2021, USA) [17]	Female/59	Chronic diarrhea and cutaneous ulcers	Duodenum	Villous atrophy	Features potentially suggestive of malabsorption (villous atrophy, chronic diarrhea, and weight loss)	Negative	Discontinuation of losartan	Resolution of symptoms and lesions
Berrocal et al. (present study, Colombia)	Female/61	Abdominal pain and chronic diarrhea	Terminal ileum	Villous shortening, intraepithelial lymphocytosis (55)*, lymphoplasmacytic infiltrate	Vitamin B12 deficiency, consistent with ileal involvement	Negative	Discontinuation of losartan	Clinical improvement

In all previously reported cases, histological evaluation demonstrated duodenal villous atrophy. In contrast, our case showed isolated ileal involvement with a histologically normal duodenum, highlighting the atypical nature of this presentation. Symptom resolution occurred after losartan withdrawal in all cases. The reason for isolated ileal involvement with sparing of the duodenum in our patient remains uncertain. Although duodenal injury is the most commonly reported manifestation of ARB-associated sprue-like enteropathy, previous reports suggest that intestinal involvement may be patchy and heterogeneously distributed throughout the small bowel, occasionally extending to the ileum and colon [18]. Therefore, a histologically normal duodenum does not necessarily exclude this entity and may reflect distal-predominant disease or sampling limitations related to the focal nature of mucosal injury.

Additionally, features suggestive of malabsorption have been reported in some cases of ARB-associated enteropathy [15-17], although this is not consistently or explicitly described across all reports. In particular, while certain cases demonstrate clear biochemical and clinical evidence of malabsorption, others only show indirect or limited findings (Table 2). This variability has also been noted in ARB-related enteropathy more broadly [19]. In our patient, vitamin B12 deficiency was the only manifestation suggestive of malabsorption, which may be explained by ileal involvement, while no other clinical or laboratory features supporting a generalized malabsorptive syndrome were identified. This finding may also represent a potential clinical clue suggesting distal small bowel involvement in atypical cases of ARB-associated sprue-like enteropathy, although its role as a screening marker remains uncertain and requires further investigation. The borderline elevation in fecal calprotectin observed in our patient may reflect low-grade intestinal mucosal inflammation associated with this entity. Although fecal calprotectin is a nonspecific marker and cannot distinguish ARB-associated sprue-like enteropathy from other causes of seronegative villous atrophy, mild elevations may occur secondary to inflammatory cell infiltration and mucosal injury [20].

This case highlights a rare and clinically relevant presentation of losartan-induced sprue-like enteropathy, characterized by isolated ileal involvement with a normal duodenum. A comprehensive diagnostic workup excluded alternative etiologies, and clinical improvement after drug withdrawal supports a causal relationship. However, some limitations should be acknowledged, including the absence of histological follow-up and the lack of HLA-DQ2/DQ8 testing, which may have further contributed to the evaluation of seronegative villous atrophy. Additionally, the diagnosis remains inferential in the absence of rechallenge, and, although an extensive evaluation was performed, not all differential diagnoses can be completely excluded. Further studies are needed to better characterize the spectrum of this condition.

## Conclusions

ARB-associated sprue-like enteropathy is a rare but reversible cause of chronic diarrhea and malabsorption. Although most reported cases involve olmesartan, increasing evidence suggests that this entity may represent a class effect of ARBs, including losartan. This case highlights an atypical presentation with isolated ileal involvement and a histologically normal duodenum, expanding the recognized spectrum of disease. Clinicians should maintain a high index of suspicion for this condition in patients receiving ARBs who present with unexplained chronic diarrhea, seronegative villous atrophy, or features of malabsorption, even in the absence of duodenal involvement, as early recognition and prompt drug withdrawal may lead to complete clinical resolution and prevent unnecessary diagnostic and therapeutic interventions.
